# Estradiol Supplement or Induced Hypertension May Attenuate the Angiotensin II Type 1 Receptor Antagonist-Promoted Renal Blood Flow Response to Graded Angiotensin II Administration in Ovariectomized Rats

**DOI:** 10.1155/2022/3223008

**Published:** 2022-07-01

**Authors:** Samira Choopani, Mehdi Nematbakhsh

**Affiliations:** ^1^Department of Physiology, Isfahan University of Medical Sciences, Isfahan, Iran; ^2^Water & Electrolytes Research Center, Isfahan University of Medical Sciences, Isfahan, Iran

## Abstract

**Backgrounds:**

Estrogen replacement therapy (ERT) and hypertension may influence females' renin-angiotensin system (RAS) and its components. The angiotensin II (Ang II) type 1 receptor (AT1R) antagonist (losartan) may promote renal blood flow (RBF), and it is widely used in the clinic to control hypertension. The main objective of this study was the effects of estradiol or induced hypertension on RBF response to Ang II in losartan-treated ovariectomized (OVX) rats.

**Methods:**

Two groups of OVX rats were treated with placebo (group 1) and estradiol (group 2) for period of four weeks, and another group of OVX rats was subjected to induce hypertension by two-kidney one clip (2K1C) model (group 3). All the groups were subjected to the surgical procedure under anesthesia, and AT1R was blocked by losartan. RBF and renal vascular resistance (RVR) responses to Ang II administration were determined and compared.

**Results:**

Mean arterial (MAP) and renal perfusion (RPP) pressures in group 3 and uterus weight (UT) in group 2 were significantly more than other groups (*P* < 0.05). Ang II infusion resulted in dose-related percentage change increase in RBF and decrease in RVR. However, these responses in the OVX-estradiol and OVX-hypertensive rats were significantly lower than in the OVX-control group (*P* < 0.05). For instance, at the dose of 1000 ng/kg/min of Ang II administration, the percentage change of RBF was 45.1 ± 10.4%, 17.9 ± 2.3%, and 16.7 ± 4.7% in the groups of 1 to 3, respectively.

**Conclusion:**

Losartan prescription in some conditions such as hypertension or ERT could worsen RBF and RVR responses to Ang II.

## 1. Introduction

High blood pressure is an important public health issue and the third leading cause of death in the world, and its incidence is expected to increase by 30% in 2025 [1]. In 2017, the American Heart Association (AHA) defined high blood pressure in adults as systolic blood pressure (BP) ≥ 130 mmHg and diastolic blood pressure (BP) ≥ 80 mmHg [2]. Therefore, the control of blood pressure is an important issue in maintaining community health, and the most common clinical intervention for controlling blood pressure is to alter the effect of renin-angiotensin system (RAS) components on hemodynamics parameters in blood circulation system.

RAS plays a critical role in regulating arterial blood pressure and body fluids [3]. Angiotensin II (Ang II) is one of the most potent biological products of RAS [3]. Most of the activity of Ang II on renal circulation and function is performed by its receptors distributed throughout the kidney. The two main groups of Ang II receptors are type I (AT1R) and type II (AT2R) [3]. AT1R and AT2R activations induce vasoconstriction and vasodilation, respectively [4]. Administration of exogenous Ang II via AT1R results in a dose-related reduction of renal blood flow (RBF) and glomerular filtration rate (GFR) [5]. Losartan is an antagonist for AT1R, and it is usually used in the clinic for hypertension controlling [6]. Although the clinical goal of losartan is to control blood pressure, the drug can potentially alter RBF [7–9], possibly by reducing renal vascular resistance (RVR). In normotensive and hypertensive animals, losartan increases RBF and induces either no change or an increase in GFR [10]. Experimental studies in the Ang II infused hypertensive rats showed that GFR was lower than normotensives one [11], which acute treatment with losartan could not fully restored it but chronic treatment with losartan prevents the decreases in GFR [11, 12]. Also, in two-kidney one clip (2K1C) Goldblatt hypertensive rats, acute administration of losartan due to the effects of severe vasodilation reduced GFR [13].

Also, it is well documented that RAS influence by sex chromosomes and gonadal hormones and exhibits sex-related differences [4, 14]. The vasodilatory effects of estrogen are mediated through various mechanisms such as nitric oxide (NO), opening of Ca^2+^-activated K^+^ channels, decrease levels of endothelin-1, Ang II, and catecholamines [14, 15]. Estrogen increases the activity of plasma renin, angiotensinogen, Ang I, and Ang II [16]. Despite the increase in RAS substrates, estrogen downregulates the AT1R and instead stimulates AT2R expression [16]. This sex hormone also increases renal ACE2 expression [16]. In female rats, the response to Ang II is mediated via AT2R [17] by an estrogen-dependent mechanism [18]. In ovariectomized (OVX) rats, AT1R expression in aortic tissue and vascular smooth muscle cells upregulated, and estrogen therapy downregulated this receptor [19]. An experimental study revealed that estrogen might shift the vasoconstrictor–vasodilator balance of the RAS [20]. Despite the conflicting evidence, the activation of Ang II–converting enzyme (ACE)/AT1R pathways may reduce by estrogen [21].

Hypertension is one of the most important causes of death and disability worldwide [22]. In hypertension, intrarenal Ang II receptors' regulation seems to be more complex because vascular and tubular Ang II receptors respond differently [23]. 2K1C is an accepted model of Ang II-induced hypertension, while intrarenal RAS has an essential role in initiating and maintaining hypertension in the 2K1C model [24].

It is documented that administration of exogenous Ang II promotes RBF when AT1R is blocked [25, 26]. However, an important question remains unanswered. Do RBF and RVR responses to Ang II in the condition of AT1R blockade alter by hypertension or estrogen? To answer this question, we investigated the effective administration of high-dose estradiol or induction of 2K1C on renal hemodynamic responses to Ang II administration in the condition of AT1R blockade in OVX rats.

## 2. Material and Method

### 2.1. Animal

Eighteen adult female Wistar rats (7-9 weeks) were housed in an animal room with a 12 h light/dark cycle, temperature 23 ± 2°C, and free access to food and water. The experimental protocols were approved by the Ethics Committee of the Isfahan University of Medical Sciences (IR.MUI.MED.REC.1397.327).

### 2.2. Study Groups

The animals were divided into three groups (6 animals in each group), and all the animals underwent surgery for ovariectomy by making a 2 cm incision in the subabdominal area. After ligation of ovarian tubes, the ovaries were removed very carefully. The muscle and skin were sutured, and the animals were placed under a heated lamp for recovery [27]. To induce hypertension in a group of rats, simultaneously with ovariectomy, a 2K1C model was applied. Accordingly, an incision was used on the right side of the abdomen, the right renal artery was isolated, and a U-shaped silver clip (lumen diameter of 0.2 mm) was placed around the renal artery to induce partial occlusion [28]. The non-2K1C (normotensive) animals also had a complete surgical intervention, just without a silver clip around the renal artery. Therefore, the groups were assigned as follows:

Group 1 (named OVX + losartan): the OVX and non-2K1C (normotensive) rats were subjected to receive sesame oil weekly as a placebo via intramuscular injections for period of four weeks

Group 2 (named OVX + Est + losartan): the OVX and non-2K1C (normotensive) rats received high-dose estradiol valerate (500 *μ*g/kg/week, Aburaihan Co., Tehran, Iran) dissolved in sesame oil via intramuscular injections for period of four weeks [27]

Group 3 (named OVX+2K1C + losartan): the OVX rats were subjected to 2K1C and received sesame oil weekly as a placebo via intramuscular injections for period of four weeks

### 2.3. Experimental Procedure

After four weeks, the rats were anesthetized with urethane (1.7 g/kg^−1^ i.p.; Merck, Germany). The trachea was cannulated to facilitate air ventilation, and polyethylene catheterization of carotid, femoral arteries, and jugular vein was performed to measure mean arterial pressure (MAP), renal perfusion pressure (RPP), and drug administration, respectively. The carotid and femoral catheters were connected to a Power Lab System (AD Instrument, Australia). An adjustable clamp was positioned around the abdominal aorta above the left renal artery to retain RPP at the control level during Ang II infusion [29].

The rats were placed in a lateral position, the left kidney was exposed, the renal artery was isolated, and an ultrasound flow probe was placed (TRANSONIC MA0.7PSB, Flow Probe, USA) around it for direct RBF measurement. The MAP, RPP, and RBF were measured continuously over the experiment. After 30 minutes and achieving a stabilization condition for the animal, AT1R antagonist (losartan) was infused via the vein catheter. The losartan was administrated at a bolus dose of 5 mg/kg followed by continuous infusion at 5 mg/kg/h. Thirty minutes after administration of antagonist, intravenous injection of Ang II at graded doses of 30, 100, 300, and 1000 ng/kg/min was infused using a microsyringe pump (New Era Pump System Inc., Farmingdale, NY, USA). Each dose was given until the response of MAP, RPP, and RBF reached a plateau (approx. 15 min). The last 3-5 minutes of each stage was used to measure MAP, RPP, and RBF. The RPP/RBF ratio was determined to express RVR. Then, the rats were sacrificed humanely, and the kidneys and uterus were weighed rapidly.

### 2.4. Statistical Analysis

Data were presented as mean ± SEM and were analyzed using the statistical software SPSS 20. One-way analysis of variance (ANOVA) was applied to baseline data. The effects of antagonist or vehicle treatments on basal variables were compared via repeated measures ANOVA. Post hoc analysis Tukey was used to determine specific effects within each group. MAP, RPP, RBF, and RVR responses to Ang II administration were compared via ANOVA for repeated measures. *P* < 0.05 was considered statistically significant.

## 3. Results

### 3.1. Baseline Measurements

**Figure 1:**
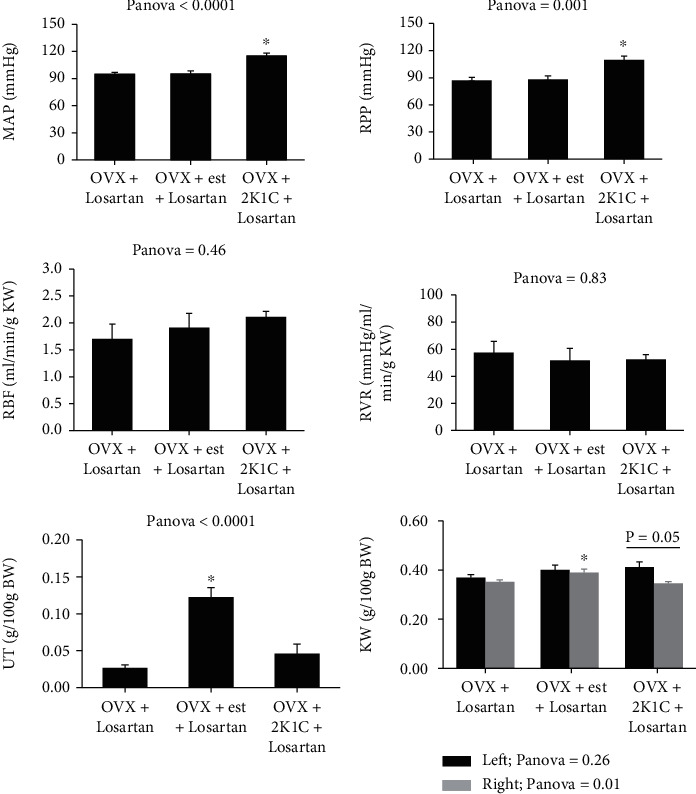
The baseline data. Mean arterial pressure (MAP), renal perfusion pressure (RPP), renal blood flow (RBF) per g left kidney weight (LKW), renal vascular resistance (RVR) per g LKW, uterus weight (UT) per 100 g of body weight (BW), and left and right kidney weight per 100 g BW. The *P* values were obtained by one-way ANOVA and Tukey as post hoc multiple comparisons. The *P* value for comparison between LKW and RKW was obtained by paired *t*-test. The star (^∗^) indicates a significant difference from other groups (*P* < 0.05).

In the equilibrium period (before administration of losartan), no significant differences were observed in basal MAP and RPP in normotensive groups (Figure 1), but in the hypertensive group (group 3), MAP and RPP were significantly increased, which confirmed the successful induction of hypertension in 2K1C rats. There was no significant difference in RBF normalized to left kidney weight (RBF/g LKW) and RVR normalized to LKW (RVR/g LKW) between the groups. Also, uterus weight per 100 g body weight (UT/100 g BW) was greater in estradiol-treated animals compared to vehicle-treated animals, which confirmed the effect of estradiol (Figure 1). There was also significant difference between left and right kidney weighs (LKW and RKW) per 100 g body weight in the 2K1C group (*P* = 0.05) which indicates the effect of clipping on right renal artery during induction of hypertension (Figure 1). It is also indicated that estradiol administration increased the RKW significantly (*P* < 0.05).

### 3.2. Effect of Antagonist

**Figure 2:**
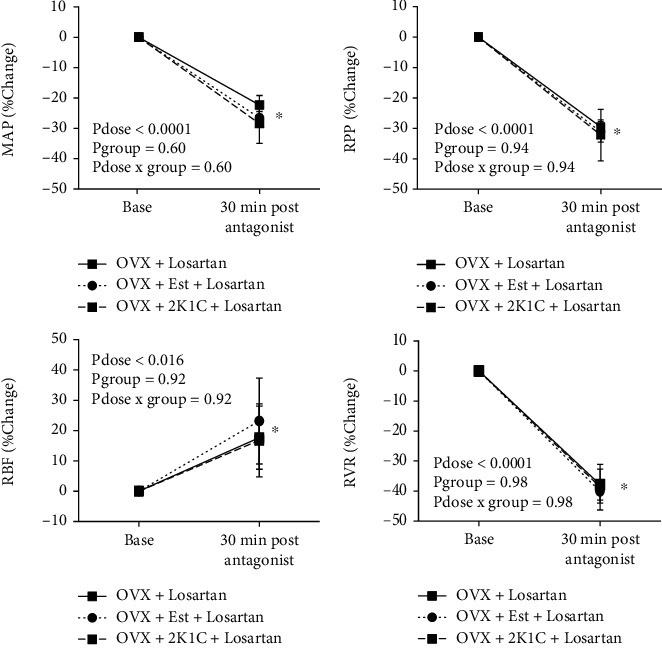
The effect of antagonist. The percentage change of mean arterial pressure (MAP), renal perfusion pressure (RPP), renal blood flow (RBF), and renal vascular resistance (RVR) at 30 min postantagonist (losartan) administration. The *P* values were obtained by ANOVA for repeated measure. The star (^∗^) indicates a significant difference from the base for all the groups (*P* < 0.05) obtained by paired *t*-test.

Before Ang II infusion, it is necessary to determine the antagonist effect on MAP, RPP, RBF, and RVR. Therefore, 30 min postantagonist administration was considered as a time for antagonist effect [25], and the hemodynamics parameters were determined. No significant differences were observed in percentage change of MAP, RPP, RBF, and RVR between the groups after administration of losartan (Figure 2). However, a significant decrease in MAP, RPP, and RVR (*P*_dose_ < 0.0001) and a significant increase of RBF (*P*_dose_ = 0.016) from the baseline were observed by losartan. For example, in groups 1 to 3, the percentage increase of RBF was 17.6 ± 10.4%, 23.1 ± 14.1%, and 16.7 ± 12%, respectively, but no significant differences were detected between the groups (*P*_group_ = 0.92). These findings revealed that losartan reduced MAP, RPP, and RVR as expected. However, it increased RBF in all the three groups.

### 3.3. Responses to Ang II Infusion

**Figure 3:**
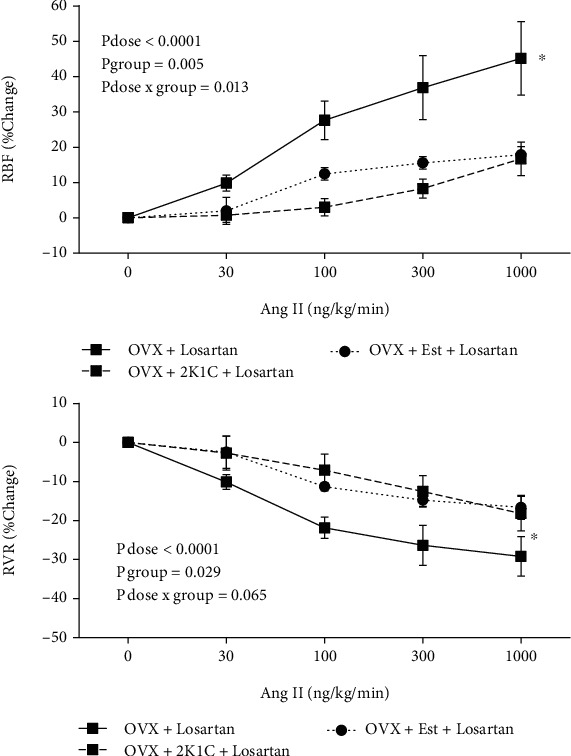
The responses to angiotensin II (Ang II) administration. The percentage change of renal blood flow (RBF) and renal vascular resistance (RVR) to graded Ang II infusion. The *P* values were obtained by ANOVA for repeated measure. The star (^∗^) indicates a significant difference from other groups (*P* < 0.05).

Ang II infusion resulted in dose-related percentage change increase in RBF and decrease in RVR in all of the groups (Figure 3). In the group 1 (OVX + losartan), as the dose of the Ang II infusion increased, the RBF and RVR responses increased. However, these responses in group 2 (OVX + Est + losartan) and group 3 (OVX+2K1C + losartan) were significantly lower than group 1 (OVX + losartan) (*P* < 0.05, Figure 3). For example, at the dose of 1000 ng/kg/min of Ang II administration, the percentage change of RBF was 45.1 ± 10.4%, 17.9 ± 2.3%, and 16.7 ± 4.7% in the groups of 1 to 3, respectively (Figure 3).

## 4. Discussion

The study's main objective was to determine the role of high-dose estradiol and induce hypertension on renal hemodynamic responses to Ang II infusion when AT1R was blocked by losartan in OVX rats. The results demonstrated that estradiol administration and 2K1C-induced hypertension attenuated the effect of losartan on RBF and RVR responses to Ang II infusion in female OVX rats.

In the present study, we observed that RBF response to Ang II infusion in group 2 (OVX + Est + losartan) was different from group 1 (OVX + losartan) due to the role of estrogen. This issue has been proven that the dose of estradiol is an important factor affecting RAS components. In physiological doses, estrogen through different signaling pathways causes vasorelaxation [30], but in this study, we used supraphysiological dose of estradiol. Laragh et al. have shown that high levels of estrogen during pregnancy or the use of oral contraceptives stimulate RAS, and it may lead to a significant increase in plasma renin level [31] followed by Ang II formation. Safari et al. reported that administration of high-dose estradiol in normotensive ovariectomized rats increases RBF and RVR responses to Ang II infusion [27], and this response was not remained when AT2R were acutely blocked with PD123319, while physiological levels of estrogen might promote the vasodilator actions of AT2R activation in the renal vasculature. So maybe supraphysiological levels of estrogen are also able to promote the vasoconstrictor actions of AT2R with an unknown mechanism. Many studies have shown that AT2R-mediated vasoconstriction has been observed under a variety of conditions, such as in the mesenteric vasculature of spontaneously hypertensive rats (SHR) in vitro [32], the rat hydronephrotic kidney [33], the kidneys of rats with heart failure [34], and the renal medullary circulation of both normal rats and rabbits [35] and rats with renovascular hypertension [36].

Another finding of the present study was that in group 3 (OVX+2K1C + losartan), RBF response to Ang II infusion was more than group 1 (OVX + losartan). Many experimental studies have shown that hypertension affects RAS components [24, 37, 38]. For example, Kim et al. showed that AT1R expression decreased slowly in both kidneys in the 2K1C model [24]. AT1R protein also was reduced in both kidneys in 2K1C hypertensive rats and Ang II-induced hypertensive rats [38]. AT1R binding was decreased in the glomeruli and the inner stripe of the outer medulla in Ang II-induced hypertensive rats, but AT1R density is maintained in the proximal tubules along with increased ACE binding [39]. Also, glomerular AT1R diminished at two weeks after clipping, but those vascular receptors did not reduce until 16 weeks in 2K1C rats [40]. So, it could be that more AT1R expression in the normotensive group than the 2K1C group causes RBF to be more in this group than the hypertensive group after losartan administration.

Another issue that might be discussed is the decreased ACE2 expression in hypertensive rats. Experimental studies proved MasR expressions remain unchanged in hypertensive rats [41] or downregulated in kidneys of SHRs [42]. ACE2 is the primary enzyme that catalyzes the conversion of Ang II to Ang 1-7, and it is reduced in spontaneous hypertensive rats (SHR) model compared with Wistar-Kyoto (WKY) rats [43]. Crackower et al. showed that ACE2 was decreased in kidneys from three separate hypertensive rat strains: salt-sensitive sabra hypertensive rats (SBH/y), SHR, and stroke-prone spontaneously hypertensive rats (SHR-SP) [44]. Prieto et al. demonstrated ACE2 mRNA levels, and its activity reduced in both kidneys of Goldblatt hypertensive rats [45]. In OVX renal-wrap rats, ACE2 activity reduced, but estrogen therapy improves ACE2 activity and protein expression [46]. Ang II also reduces the expression of ACE2 in the heart, kidneys, and astrocytes [47–49]. On the other hand, the decrease of ACE2 expression exacerbates hypertension [46]. Accordingly, decrease activation of ACE2/MasR pathway also is another reason that affects RBF response to Ang II in the OVX+2K1C + losartan group.

Finally, we had some limitation in this study. We did not investigate the expression of the receptors and the dosage of ACE. Having this information may provide a more comprehensive interpretation of the results.

## 5. Conclusion

Hypertension and female sex hormones influenced RAS components. Administration of losartan increases RBF, but 2K1C induced hypertension possibly by a decrease of AT1R expression, as well as, reduce activation of ACE2/MasR pathway, and supraphysiological doses of estrogen to promote the vasoconstrictor actions of AT2R with an unknown mechanism could increase RBF and RVR responses to Ang II infusion.

## Data Availability

Data will be available by request.
